# Editorial

**Published:** 1984-04

**Authors:** 


### Editorial

OUT OF THE FRYING PAN—
Surmount one obstacle and you are faced with the next. Lack of material has in the past been a problem, now this has been overcome we have to pay for a larger *Journal* out of the subscriptions of a static Society. We have had some support from advertisers and in 1982 two generous gifts from drug firms of £1000 each from Smith, Kline and French and from Farmitalia. The majority of our members are general practitioners and though it is our aim to provide reading of general interest and some of special interest to general practitioners nevertheless the bulk of our reading matter consists of specialist articles and reports of specialist societies of special interest to the hospital community. It seems right therefore to seek more support for our *Journal* from the three Bristol Hospitals. Though nothing is as yet decided, our overtures are being sympathetically considered and we hope to be able to announce in our next issue that, without any further increase in subscription rates we are
OUT OF THE FIRE.

Dr. COLIN TRIBE
Dr. Tribe died, sadly, at an early age and his Obituary appears below. He was our deputy editor and also one of our regular correspondents. His last contribution, written only a few days before his death 'Anastomosing Drains' is a little gem in the comic vein. (January 1984, p 24).

Dr. Brown's obituary gives us some idea of this diversely talented and lovable man but only those who caught the twinkle in his eye and heard his explosive laugh really knew what he was like.

We are delighted that Dr. J. D. Davies has agreed to take over his corresponding role and suspect he is a bird of the same feather. Can it be that pathologists, of all men most continuously aware of death are those most determined to extract what life and living have to offer? This was most certainly true of Colin Tribe.

